# From complex social interventions to interventions in complex social systems: Future directions and unresolved questions for intervention development and evaluation

**DOI:** 10.1177/1356389018803219

**Published:** 2018-10-31

**Authors:** Graham F. Moore, Rhiannon E. Evans, Jemma Hawkins, Hannah Littlecott, G.J. Melendez-Torres, Chris Bonell, Simon Murphy

**Affiliations:** Cardiff University, UK; Cardiff University, UK; Cardiff University, UK; Cardiff University, UK; Cardiff University, UK; London School of Hygiene & Tropical Medicine, UK; Cardiff University, UK

**Keywords:** complex interventions, complex systems, evaluation, methodology, population health, social intervention

## Abstract

Complex systems approaches to social intervention research are increasingly advocated. However, there have been few attempts to consider how models of intervention science, such as the UK’s Medical Research Council complex interventions framework, might be reframed through a complex systems lens. This article identifies some key areas in which this framework might be reconceptualized, and a number of priority areas where further development is needed if alignment with a systems perspective is to be achieved. We argue that a complex systems perspective broadens the parameters of ‘relevant’ evidence and theory for intervention development, before discussing challenges in defining feasibility in dynamic terms. We argue that whole systems evaluations may be neither attainable, nor necessary; acknowledgment of complexity does not mean that evaluations must be complex, or investigate all facets of complexity. However, a systems lens may add value to evaluation design through guiding identification of key uncertainties, and informing decisions such as timings of follow-up assessments.

## Background

The UK’s Medical Research Council (MRC) guidance for the development and evaluation of complex interventions describes iterative phases of intervention development, feasibility testing, effectiveness evaluation, and wider implementation (Craig et al., 2008a). Building on earlier guidance (Campbell et al., 2000), it has substantially influenced the design, conduct, funding and reporting of public health intervention research. Nevertheless, the decade since its publication has seen substantial critiques. In particular, a growing literature has advocated movement toward complex systems approaches to social intervention research (Brainard and Hunter, 2016; Hawe et al., 2009; Howarth et al., 2016; Moore and Evans, 2017; Rutter et al., 2017; Shiell et al., 2008). However, this advocacy, has been accompanied by fewer efforts to articulate what this might look like in practice (Carey et al., 2015; Greenwood-Lee et al., 2016; Luke and Stamatakis, 2012). For example, new guidance on taking account of context in population health intervention research concludes that ‘a comprehensive understanding of interventions in context implies the adoption of a systems approach’ (Craig et al., 2018: 26). However, the authors argue that systems rhetoric is rarely operationalized in a manner which generates useful evidence, offering limited reflection on what such a perspective might mean for the development and evaluation of interventions in complex social systems.

While beyond the remit of a single paper to address all of these vexed challenges, we reflect on some ways in which the 2008 MRC framework, which is currently undergoing revision, might be re-conceptualized through a complex systems lens. We begin by operationalizing Hawe et al.’s (2009) concept of interventions as ‘events within systems’ which aim to disrupt the functioning of complex systems through changing relationships, displacing entrenched practices, and redistributing and transforming resources. We do this by reframing a range of recent public health interventions from this perspective. We draw on illustrative examples from a range of settings, with a particular emphasis on schools.

We then consider the implications of this perspective for each phase of the MRC framework in turn. We argue first for the development of social interventions to look beyond a focus on specific tangible products, and toward a broader goal of understanding system dynamics, and modelling strategies for their disruption. We then highlight key challenges in relation to conceptualization of feasibility and acceptability as dynamic concepts, which may shift positively or negatively over time in response to feedback. We argue that a ‘whole systems’ perspective to evaluation may be unattainable; it is never possible, nor perhaps even desirable, for any one evaluation to investigate all aspects of complexity arising from a system change. However, a complex social systems lens may play an important role in shaping questions posed of interventions, and interpretation of evaluation data.

## What is a complex social intervention?

Recent years have seen much debate about where complexity resides in social intervention research (Moore et al., 2017). Original MRC guidance (Campbell et al., 2000) located complexity within the components of an intervention, contrasting ‘complex’ multi-component programmes with ‘simple’ mono-component drug therapies. But an intervention composed of multiple components may not necessarily be complex, but merely complicated. Illustrating this distinction, Glouberman and Zimmerman (2002) argue that sending a rocket to the moon is *complicated.* It requires great skill and numerous interacting components. However, it can be divided into discrete sets of actions with predictable, stable and linear consequences. Raising a child by contrast is *complex*, due to the emergent, unpredictable, and non-linear nature of associations between actions and outcomes. While a complicated machine such as a rocket is passively acted upon by human actors, children and parents are active agents, whose behaviour continuously adapts in response to feedback from one another, generating patterns of behaviour for the family as a whole. Actions of parents cannot be easily isolated from broader family and community systems (Bronfenbrenner and Bronfenbrenner, 2009). Individual parenting practices generate variable impacts across time and space, and cannot be easily isolated from the holistic work of parenting. Social interventions within families are therefore complex primarily due to the social systems within which these actions occur, the contextually contingent nature of impacts, and the agency of the groups and individuals whose behaviours they aim to influence.

## What is a complex social system?

Definitions of complex social systems within the literature vary. For example, defining schools as complex social systems, Keshavarz et al. (2010) emphasize the extent to which their functioning is shaped by interactions among a diverse range of ever changing actors, such as staff, pupils and parents. These interactions occur within and between activity settings (time-space bound patterns of social interaction) such as school classes, or parent-teacher association meetings (Hawe et al., 2009). Schools are nested within educational supra-systems. They have autonomy, but within constraints imposed by external systems. Schools constantly evolve in response to external pressures, new technologies or techniques, and changes in perceptions as to which skills young people need to navigate their current and future lives. Drawing upon concepts from the complexity literature, Chandler et al. (2016) emphasize dimensions of system complexity such as self-organization and system histories as critical in understanding the introduction of changes such as new surgical practices in hospitals. The functioning of systems such as hospitals, from this perspective, is not centrally determined but self-organized through the collective actions of the agents within it. Chandler and colleagues (2016) argue that where particular surgical practices are habituated, attempts to introduce change will be met by self-organization processes which wash the change out of the system (Hawe et al., 2009). Hence, in face of a disruptive intervention, system stability is ensured through maintenance of the status quo, rather than assimilation of intervention into the system.

Schools and hospitals represent examples of bounded ecological systems, via which many social interventions to improve population health are delivered. However, in turn, these bounded social systems are a part of broader networks of agents, whose interactions influence health. For example, the youth tobacco system includes industry, retailers, scientists, public health professionals, governments, media, communities, schools, families and children; the dynamic interplay among these groups of actors serves to maintain or disrupt the status quo in smoking prevalence over time (National Cancer Institute, 2007). While there is further work to be done in unifying conceptual thinking in relation to complex systems theories, and their application to social interventions in health, some key concepts are summarized in Table 1 (Keshavarz et al., 2010; Mitchell, 2009; Rickles et al., 2007), illustrated through the example of schools.

**Table 1.** Some key characteristics of complex social systems.

System characteristic	Illustrative example
The functioning of complex social systems is shaped by patterns of ***interaction among diverse and ever changing agents*** Complex social systems typically comprise nested sub-systems and are part of larger supra-systems; Systems have permeable boundaries and hence influential interactions occur both ***within*** and ***between complex social systems***; System survival depends upon ***ceaseless adaptation***, with systems responding to changes to internal and surrounding system structures; Systems typically have a combination of formal **rules**, and more informal **‘ethos’**, or shared understandings about the norms and values of the system; New ways of working give rise to ***feedback loops*** that reinforce system behaviour, or lead to discontinuance; Systems have a propensity toward ***self-organization***, with order emerging through spontaneous interactions of agents within the system, rather than central planning. Efforts to introduce change create disruption, triggering agents to self-organize to return the system to an ***attractor*** state (i.e. a new state of relative stability); ***System histories*** and ***starting points*** play an important role in shaping responses to a new innovation.	School teachers and staff constantly change within a school, although a degree of functionality is maintained; School classes (sub-system) and national education systems (supra-systems); Schools interact with one another and with their local neighbourhoods to influence pupils’ education and health; The content and format of teaching constantly evolves in relation to external changes; Schools have formal policies and mission statements, but also more informal understandings of norms for staff and student conduct; Schools adjust their policies and practices on the basis of feedback from regulatory authorities, students and parents; School staff work collectively to find the most efficient route to achieving a system’s primary goals, which may involve, for example, de-prioritizing anything seen as peripheral to core business of educational attainment Following disruption, self-organization processes return the system to a state of stability. This may be achieved through assimilating a new way of working into the everyday functioning of the system, or through washing it out and returning to pre-intervention functioning; New teaching practices which are consistent with the history of the system may be accommodated more readily than those which represent fundamental changes in direction.

## From ‘complex social interventions’ to ‘events’ within complex social systems

Hawe and colleagues (2009) argue that public health interventions should be viewed not as sets of decontextualized components, but as ‘events’ within complex social systems. Viewing interventions in this way takes us away from traditional attempts to describe new ways of working in isolation from the systems they attempt to change. For example, a surgical intervention may involve installation of a new component (e.g. a transplanted organ) into a complex biological system (the human body). However, the intervention is not just the organ, but includes also the surgical process, and the interaction of the new organ with this dynamic system. Likewise, framing social interventions as an attempt to *change* a system’s dynamics, intervention can only be defined with reference to the system dynamics it attempts to disrupt.

Smoke-free legislation is an example of an upstream public health intervention which can be thought of as a critical event within the history of the tobacco control system. Prior to legislation, communication of emerging science on harms of second-hand smoking reframed public discourse in a manner which countered civil liberties objections (Chapman, 2008). The system moved toward a tipping point (i.e. the point in the history of a system at which the right configuration of context and mechanisms creates the conditions for change (Mitchell, 2009; Pawson, 2006)), at which legislation once thought authoritarian and illiberal was now embraced (Holliday et al., 2009). This legislation was attributed with reducing second-hand smoke exposure among hospitality staff (Semple et al., 2007) and growing adoption of smoke-free homes (Jarvis et al., 2009), generating feedback loops which acted as inputs for further action. The acceptability of smoking in front of non-smokers continued to decline, as advocacy turned toward child protection (Chapman, 2008). Bans on smoking in cars carrying children followed (Moore et al., 2015b). Hence, this ‘event’ was associated with change partly because it occurred at a critical time in the history of the system, in turn playing a part in activating changes in system dynamics which paved the way for further action.

While systems perspectives are often discussed as relevant to transformative upstream changes such as smoke-free legislation, a straightforward dichotomy between individual-level, and system-level interventions is unhelpful. The complexity of social systems, and interventions within them, can perhaps more accurately be described as a continuum. As described, the family is a complex system; nevertheless, the nature of families perhaps has more in common across time and space than do many more highly complex social systems such as schools. Hence, while some parenting interventions have not translated well between different systems (Robling et al., 2016), others have been able to achieve similarly positive disruptions to family functioning across varying international health and social care systems (Gardner et al., 2016; Leijten et al., 2016).

Indeed, even apparently very simple downstream social interventions can be conceived as attempts to disrupt existing system dynamics. For example, in a recent trial, Aveyard and colleagues (2016) found significant effects of a brief GP intervention to encourage patients to engage with weight loss services. In traditional terms, this could be defined as a combination of behaviour change techniques, adherence to which triggers change. However, a systems perspective reconceptualizes the doctor–patient consultation as a micro-system, or activity setting (Hawe et al., 2009). The intervention focuses on one small part of a broader healthcare system, the doctor–patient interaction. However, these patterns of interaction are bound in the history of the system. Patients’ trust in physicians as a primary source of health information is situated in the interactions between actors in a particular cultural and historical context (Hall et al., 2001). Intervention assumes that there is something sub-optimal in the nature of interactions between doctors and patients, and that change can be activated through systematically altering a small number of discrete practices.

For interventions at the simpler, more downstream, end of this continuum, the conditions necessary for optimal functioning may be more stable across time and space than for more transformative system changes, such as smoking legislation. Hence, while accommodating even seemingly simple discrete practices will require an understanding of how the system functions, and how to alter it, the dominant emphasis within the literature on individual-level behavioural change processes in understanding such interventions is perhaps appropriate (Michie et al., 2013). However, a focus on individual-level behaviour change processes becomes increasingly inadequate in understanding how changes at higher system levels influence population health outcomes (Moore and Evans, 2017).

## What does a systems lens mean for intervention development and evaluation?

New guidance on accounting for the role of context throughout the phases of the MRC framework (Craig et al., 2018) concludes that comprehensively understanding interventions in contexts implies a systems perspective. To date, much intervention research, informed by models such as the 2008 MRC framework, has targeted easily modifiable elements of complex systems, which are at best minimally disruptive of system functioning (Hawe, 2015). Key benefits of a systems perspective may lie in the extent to which it draws focus beyond minimally disruptive interventions, and toward more disruptive system changes operating at multiple system levels. A systems lens also draws our attention to a need to consider the ramifications of changing certain parts of a complex social system for outcomes produced by the system as a whole, throughout intervention development and evaluation. For the remainder of this article, we reflect on some key areas where changes to dominant practices in intervention research may be necessary if greater alignment with a systems perspective is to be achieved (see Table 2).

**Table 2.** Stages of the MRC framework, and considerations from a traditional, or complex social systems, perspective.

Stage	Traditional stage focus	Additional considerations from an events in systems perspective
Intervention development	Identification of intervention components which have been effective in addressing similar problems elsewhere; Theorization of how components interact with one another to influence the target outcome.	Identification of how the dynamics of a particular social system perpetuate and sustain sub-optimal health outcomes; Theorization of how patterns of system behaviour might be disrupted by the introduction of new ways of working to optimize the health promoting potentials of the system.
Feasibility and pilot testing	Exploration of whether an intervention, and evaluation, approach is acceptable to key stakeholders; Short-term testing of whether an intervention can be feasibly implemented with fidelity and acceptability to participants; Refinement and testing of key methodological parameters.	Feasibility and acceptability as dynamic concepts, changing over time through positive reinforcing or balancing feedback loops; Focus on potential of an intervention to gain traction within its system; Assessment of whether intervention can be implemented with fidelity of functions (rather than form) across a purposive range of settings. Exploration of metrics for describing systems and assessment of how to sample these in a larger study of effects.
Evaluation	Testing of the extent to which an intervention ‘works’ and is cost-effective; Process evaluation focused on whether the intervention is implemented as intended to ensure the internal validity of outcomes evaluation.	Testing and refinements of theories about: mechanisms of disruption; intended and unintended proximal and distal consequences; and system-context moderation of these; Explicit consideration of likely outcomes over time to guide durations of follow-ups. Assessment of whether intervention was implemented with fidelity of functions (rather than form) to assess the internal validity of outcomes evaluation.
Implementation	Dissemination of research evidence to key stakeholders; Maintenance of effects from evaluation in routine practice.	Implementation, in system change terms, as a process which is understood on increasing scales throughout intervention development, feasibility assessment and evaluation.

### Intervention development

In recent years an array of guidance for intervention development has emerged (Michie et al., 2011; Wight et al., 2016). Changes to funding systems, such as the MRC Public Health Intervention Development funding stream in the UK, have signalled recognition that disappointing outcomes of public health interventions in many cases arise from a tendency to rush to expensive evaluations of under-developed interventions. MRC guidance described intervention development as comprising identification of i) the evidence base, ii) appropriate theory and iii) modelling processes and outcomes. In this section, we argue that these aims are defined sufficiently loosely that they can be interpreted in ways which are consistent, or inconsistent, with a systems perspective. This depends largely on whether interventions are defined purely as a new set of components, or as a process of disrupting system functioning.

#### Identifying the evidence base: What evidence matters in understanding how to disrupt system functioning?

MRC guidance emphasized the role of evidence, particularly systematic reviews, in informing intervention development. However, while evidence provides a history of what has worked elsewhere, it provides an imprecise guide to future success (Bonell et al., 2012). Intervention effects are always relative; the implicit question of ‘how much better or worse is this way of working, compared to whatever would have been happening anyway, in this context’ is often reduced to an absolute question about ‘effectiveness’. The evidence synthesis world is rapidly developing methods for more contextually sensitive syntheses in recognition of the need to better understand transferability between differing local and international healthcare (and other) systems (Pfadenhauer et al., 2017; Petticrew et al., (in press); Booth et al., (in press)). However, a systems lens compels us to look beyond viewing the system as background noise, and toward engaging with a broader range of evidence focused on the functioning of those systems we seek to change (Petticrew et al., 2017). While new broad-based evidence synthesis processes may not be feasible for every new intervention, there is a need for involvement of academics and other stakeholders with intimate knowledge of the relevant evidence base in the process of developing new interventions. For example, interdisciplinary working with educationalists with up to date understandings of school systems is likely to be vital in avoiding the development of school health interventions that are never likely to be implementable or effective within these crowded and rapidly changing systems.

#### Identifying or developing appropriate theory: Whose theory matters in understanding how to disrupt system functioning?

MRC guidance highlights the importance of identifying or developing appropriate theory in informing intervention development. ‘Theory’, while often reduced to formal academic theory, encompasses any set of causal assumptions surrounding how defined actions produce defined consequences that can be tested using empirical data, whether derived from evidence, experience, common sense or ideology. All deliberate system changes are founded on a theory of change. The common presumption that academic theories will inherently prove superior to theories held by those with intimate knowledge of complex social systems is contradicted by the disappointing effects of many interventions based on social science theory (Prestwich et al., 2014). Many interventions developed with minimal academic input have been assimilated into everyday practice following evidence of effects. The Primary School Free Breakfast Initiative and the National Exercise Referral Scheme (Murphy et al., 2011, 2012) continue to be delivered nationwide 10 years on from evaluation.

The optimal balance of academic theory, and more contextualized and system-led theories of change, remains open to debate. A tendency for intervention development to be led from within academia, driven by an imperative to generate evidence with the greatest level of certainty, has arguably privileged minimally disruptive interventions which can be tested with greater certainty. Recent intervention development frameworks have differed in their focus on academic theory, or on contextually informed theory, as a primary guide for intervention development. The Behaviour Change Wheel (Michie et al., 2011) for example, adopts a more strongly academic psychological perspective to intervention development focused on the behaviour change functions of intervention components. By contrast, Six Steps in Quality Intervention Development (Wight et al. 2016; 6SQUID) argues that interventions ‘exert their influence by changing relationships, displacing existing activities and redistributing and transforming resources’, highlighting inter-disciplinarity and co-production as central to achieving balance between historical evidence, and ecological fit. Co-production has been central to newer case study exemplars of the development and optimization of interventions within school systems (Hawkins et al., 2017). This resonates with MRC guidance, which suggests that ‘appropriate “users” should be involved at all stages … as this is likely to result in better, more relevant science and a higher chance of producing implementable data’ (Craig et al., 2008a). Increasing emphasis on involvement of actors at multiple levels of the system signals recognition of the importance of harnessing theories from within the system, rather than purely imposing theories from without.

#### Modelling processes and outcomes (in context)

Updated MRC guidance placed emphasis on modelling intervention processes and outcomes, after consideration of relevant theory and evidence; a range of modelling approaches and their potential alignment with a systems perspective is presented in Table 3 below. Logic models are widely advocated as a means of graphically depicting the causal logic of interventions (Kellogg Foundation, 2004). These are helpful in the development of shared understandings about core mechanisms and assumptions, and to focus inquiry on key aspects of uncertainty. They have, however, tended to be linear, simplistic representations of complex realities where causal directions are described but underlying mechanisms are not (Funnell and Rogers, 2011). Hence, while an understanding of intended pathways is a useful starting point, it is also useful to consider alternative scenarios (Bonell et al., 2014b) based upon a range of assumptions about how actors within the system will respond to intervention. For example, when developing interventions concerning school food policies, one might start with a simple linear model of how these may reduce young people’s consumption of certain foods. However, experiences of actors within the school system, and the extant literature may lead us to theorize that actors, such as parents and children themselves, may respond by subverting such moves (Fletcher et al., 2014). Explicitly considering a range of alternative scenarios, with input from key actors within the system, can provide a starting point for implementation strategies, and identification of priorities for evaluation. From a systems perspective, this stage is not necessarily about specifying the precise form that a new intervention might be expected to take in every single context, but might instead be about specifying the functions of key intervention mechanisms in disrupting common patterns of system behaviour (Hawe et al., 2004).

**Table 3.** Approaches to modelling intervention processes and outcomes.

		Description of method	Applications to intervention development in complex systems	Limitations
**Logic model development**		Development of shared understandings about core processes and assumptions, and to focus inquiry on key aspects of uncertainty.	Specification of functions of key intervention processes in disrupting common patterns of system behaviour, while considering a range of alternative scenarios.	Logic models are often linear, thus over-simplify complex social processes.
**Social network analysis**	**Whole network analysis**	Modelling the structure of social ties within a whole bounded social network; Each individual in the network is asked to report who they interact with and this information is combined to analyse the overall structure.	Identification of key leverage points, and individuals whose behaviour may disproportionately influence the functioning of the system as a whole.	Requires the imposition of artificial boundaries around social interactions.
**Ego network analysis**		Development of an understanding of a social network from one individual’s perspective;	Visual elicitation within qualitative interviews that discuss how social interactions occur within a system; Allows for the permeability of systems such as schools, enabling mapping of influential relationships with outside agencies.	Understands a network from an individual’s perspective and it may, therefore, be difficult to build up an accurate picture of the whole system.
**Agent-Based Modelling**		Computer based simulations of the consequences of altering a discrete feature of a complex system for the functioning of the system as a whole; Individuals represented as active agents, whose interactions shape, and are shaped by, the functioning of the simulated system they inhabit.	Facilitates understanding of the consequences of introducing change to discrete events within systems, such as changing a feature of the physical environment; Can be used to theorize the likely nature of non-linear change processes and unintended impacts.	Modelled upon highly simplified versions of reality; Outputs highly sensitive to the validity of assumptions regarding system starting points.

An increasing number of authors advocate going beyond qualitative logic modelling processes, and toward use of systems science methods such as social network analysis (SNA) (Hawe and Ghali, 2007) and agent-based models (ABM) (Greenwood-Lee et al., 2016) to understand the dynamics of complex social systems, and model likely impacts of change. SNA has a relatively long history in public health, and has been used to understand the dynamics of peer smoking and identify intervention points to interrupt youth smoking uptake (Campbell et al., 2008). Ego network analysis, whereby a social network is mapped from the perspective of one individual, presents potential for visual elicitation within qualitative interviews. This involves the interviewee mapping their ego network within an interview, and using this visualization as a prompt to elaborate upon how social interactions occur within a system (Littlecott, 2016). Simulation approaches such as ABM have been used to model processes underpinning the social contagion of alcohol consumption (Gorman et al., 2006), and emergence of neighbourhood inequalities in health behaviours (Speybroeck et al., 2013). In ABMs, individuals represent active agents, whose interactions shape, and are shaped by, the functioning of the simulated system they inhabit. The consequences of introducing change to discrete aspects of the system, such as changing the location of a bar (Gorman et al., 2006), or assumptions about movements between rich and poor neighbourhoods are then modelled (Speybroeck et al., 2013), and can be used to theorize the likely nature of changes over time and unintended impacts. Forms of simulation models are gaining traction in fields such as alcohol policy where experimentation is more challenging to achieve. However, ABMs, which arguably offer the greatest congruence with a systems perspective, have not been widely used in public health intervention development (Speybroeck et al., 2013).

Of course, even these complex methods present simplified versions of reality. SNA requires imposition of artificial system boundaries; at some point, even the most complex network analysis will be hampered by its ignorance to potentially influential interactions beyond those boundaries. ABMs will only ever be as good as the understandings of system starting points that provide their inputs. Nevertheless, their value may lie largely in the extent to which they force intervention developers to focus carefully on constructing a clear understanding of current system functioning, before considering the impact of introducing change.

### Feasibility and pilot testing

MRC guidance argues that movement to evaluation without understanding feasibility can lead to evaluation failure, for example, due to underestimation of challenges such as recruitment (Simpson et al., 2015). Hence, guidance advocates careful feasibility and pilot testing (Campbell et al., 2000; Craig et al., 2008a). In many cases, evaluation without feasibility testing may be forced by policy timescales (Murphy et al., 2011, 2012); such evaluation remains important, not least to capture unintended harms (Ogilvie et al., 2011). Nevertheless, there remain convincing justifications for not leaping straight into a large-scale evaluation without good reason to believe it is warranted.

However, in this section we highlight some potentially perverse consequences of the drive for establishing feasibility prior to effectiveness, including the privileging of easy to implement interventions that target system points with minimal leverage. In moving away from superficial system changes, we argue that there is a need to conceive feasibility in more dynamic terms. We also revisit some key methodological recommendations, such as the use of pilot data to estimate likely effect sizes, through a systems lens.

#### Intervention feasibility: Progression criteria

While guidance for feasibility and pilot studies has commonly focused on methodological uncertainties for evaluation design,^1^ questions regarding whether an intervention is feasible are commonly a focus at this stage (Hawkins et al., 2017; Moore et al., 2013). Such questions are often posed in a binary manner, focusing on whether an intervention is delivered with fidelity in a sufficient number of sites, or if a sufficient number of stakeholders deem the intervention acceptable. Movement toward pre-specifying objective progression criteria (Avery et al., 2017) perhaps reinforces this. Such criteria are important in limiting post-hoc rationalization; without them, feasibility data can usually be interpreted as providing support for stopping or continuing. However, they often have a limited scientific basis, and there are risks in treating them as definitive evidence that a future evaluation can, or should, be conducted.

A systems lens draws our attention to the need to define feasibility and acceptability as dynamic concepts. As described, complex social systems have a strong propensity toward self-organization (Chandler et al., 2016); resistance to the introduction of a disruptive change is to be expected. However, perceptions of any new way of working are likely to change over time as the system begins to generate feedback loops. These may be positive reinforcing, leading to increasingly positive perceptions and adoption, or balancing, leading to reductions in use or discontinuance. Many system changes once viewed as ‘unacceptable’ or ‘infeasible’ have become uncontroversial norms over time; evidence based medicine was once heavily resisted as a threat to clinical freedoms (Davies et al., 2000). The assumption that, if an intervention cannot be fully implemented within the short duration of a feasibility study, it is not feasible may exacerbate the tendency highlighted by Hawe (2015) for evaluation of ‘minimally disruptive’ interventions which can be rapidly accommodated into practice, but have an insufficient impact on the functioning of the system to achieve intended outcomes.

Whole-system change interventions, such as those underpinned by the WHO Health Promoting Schools framework have shown promise in many trials, although often suffer significant implementation shortcomings, perhaps because the length of time required to implement these kinds of whole-system changes is often underestimated (Langford et al., 2015). In a recent trial of one school and community based obesity prevention intervention, intended changes to school environments took the full 3.5 years of the study to be realized (Waters et al., 2017). Hence, while perhaps more appropriate for interventions based on more discrete and relatively simple system changes, for more holistic upstream interventions, making a judgement on whether to proceed to evaluation based upon whether implementation occurred as intended within the short life cycle of a feasibility study sets them up for failure. However, postponing evaluation for several additional years is likely to be equally problematic.

Hence, as researchers and funders consider more upstream interventions, there is perhaps a need for more innovative thinking about progression to evaluation. Feasibility studies could move away from asking whether an intervention was fully delivered during the feasibility period, toward a more temporal focus on system responses to intervention, focused on how a proposed new way of working begins to gain traction within its context over time (Hawe et al., 2009). Further work beyond the scope of this article is needed in order to fully consider what ‘progression criteria’ might look like for more complex system changes.

#### Intervention feasibility: Adaptation between contexts

Within drug trials, Phase II studies typically focus on highly controlled intervention with homogeneous groups of participants to establish efficacy, before moving to assess effectiveness. Intervention is assumed to have the power to alter an outcome, but this effect may be diluted by non-ideal delivery. Likewise, original MRC guidance (Campbell et al., 2000; Medical Research Council, 2000) cautioned against allowing an intervention to evolve once evaluation had begun, arguing that this may render findings unusable. While with a drug, one can be confident that it will not morph into something else as it diffuses across contexts, a view of interventions as fixed and rigidly standardized is problematic when we view interventions as attempts to alter social dynamics within complex social systems (Hawe et al., 2004). The dynamics of systems such as schools differ substantially, and exact actions required to orient them toward healthier outcomes will differ, even where a common and coherent underlying logic is relevant across sites. An intervention that remains static while the systems surround it adapt will become redundant, and wash out of the system. While updated MRC guidance highlighted the extent to which tailoring to context is *permitted* as a dimension of complexity (Craig et al., 2008a), adaptation is arguably part of the process of accommodating a new way of working into a complex system, rather than something that requires permission.

At this stage therefore, a systems lens draws our attention to a focus on enhancing confidence that the logic underlying a proposed course of action, in terms of its key functions, is relevant to the causes of the problem, and can be replicated across a diverse range of local settings. Maximum variation sampling of case study sites may enable refinement of definitions of what it means to deliver an intervention with fidelity, in terms of how judgements will be made regarding whether differences between settings reflect adaptive tailoring, or departures from intervention logic (Van Urk, 2017) This requires careful thought about the metrics used for such sampling, in order to describe contexts in ways which meaningfully capture diversity between systems. There are of course also risks in moving toward functional definitions of fidelity which need to be carefully considered; where there is empirical uncertainty or a lack of consensus on intervention functions for example, alterations made in the name of adaptive local tailoring may inadvertently undermine functionality (Mihalic, 2004; Segrott et al., 2014).

#### Methodological parameters: Estimating sample size, recruitment and retention

Within original MRC guidance, the exploratory trial phase was expected to ‘provide unique evidence of intervention effects for the purposes of calculating power of a main larger trial’ (Campbell et al., 2000, Medical Research Council, 2000). Updated guidance argued that estimates from pilot studies must be treated with caution, though paradoxically retained ‘safe assumptions about effect sizes’ as a progression criterion (Craig et al., 2008a, 2008b). Whether feasibility trials should aim to generate an estimate of the potential effect of an intervention remains controversial. From a systems perspective, we would argue that it should not. Assumptions that effects observed in exploratory research will be borne out in full-scale evaluation are rarely upheld by the extant literature (Crawford et al., 2016). Powering a trial on pilot data is unreliable partly because the sample will be too small. From a systems perspective, additional risks include that intervention will not be sufficiently integrated with its system during a short-term feasibility pilot, while samples are likely to represent a diverse range of system starting points, rather than being a representative sample of the population to which an estimate of average effect could be extrapolated. Hence, assuming that an estimate from a pilot trial is a good estimate of likely population effect will almost certainly lead to incorrectly powered evaluations (Westlund and Stuart, 2017). Treating estimates as a signal of the likely long-term effect also risks early abandonment of interventions with long-term potential. Full-scale evaluation should ideally be powered on the basis of providing the ability to detect a clinically or sociologically meaningful change in intervention outcomes, rather than a flawed estimate from pilot data. Of course, this argument applies to efforts to estimate methodological parameters such as recruitment and retention rates within pilot studies; one should not assume that the same rates will be achieved in a full evaluation. This stage however provides valuable insights into whether viable rates can be obtained across a diverse number of settings. A decision not to proceed to full evaluation may be made for example due to failure to demonstrate that recruitment can be achieved in particular settings, such as lower socio-economic status schools or neighbourhoods. New MRC-NIHR funded guidance on feasibility studies is in development (Hallingberg et al., 2018; Moore et al., 2018), and will likely stimulate further debate and changes in practice around some of the issues identified in this article.

### Evaluation

Within original MRC guidance, all early phases build to an RCT aimed at testing if a fully standardized intervention works. Updated guidance departed from this in two important ways. First, it acknowledged that RCTs are often infeasible (Craig et al., 2008a), with guidance on the use of natural experiments following shortly after (Craig et al., 2012). Second, it signalled recognition that process evaluation was highly valuable alongside outcomes evaluation, with greater recognition of the contingency of intervention effects on context. Additional guidance for process evaluation was subsequently published (Moore et al., 2014, 2015a). In this section, we argue that no evaluation will ever be able to address the almost infinite number of uncertainties posed by the introduction of change into a complex system. Adoption of a systems lens may however, drive the focus of evaluation (i.e. which of the multitude of uncertainties posed by interventions in complex systems do we need answers to in order to make decisions, or move the field forward), and will shape the interpretation of process and outcomes data.

#### Evaluating outcomes

While the complex nature of the social world has led many to argue that methods such as RCTs are rarely useful in complex social systems (Byrne, 2011; Macintyre and Petticrew, 2000), others have defended their use within a complex systems framework (Hawe et al., 2004). Trials are the most internally valid means of establishing how much change occurred after intervention (relative to a comparator) where an intervention can be offered to individuals, or bounded units such as schools, and there is reasonable confidence that ‘treated’ individuals or units do not exert influence on ‘untreated’ cases (Rubin, 1990). Where these pre-requisites cannot be met, other methods may be preferable (Bonell et al., 2009), such as quasi-experimental studies or interrupted-time series designs. Regardless, a systems lens requires that such evaluations are designed, and their outputs interpreted, in more nuanced ways.

Impacts of system changes take time to emerge, as feedback loops build over time. For example, cycle safety measures may lead to small initial increases (Petticrew et al., in press) in cycling, which lead in turn to larger increases, as initial road safety increases are intensified by the ‘safety in numbers’ effect of a growing number of cyclists (Elvik and Bjørnskau, 2017). A short-term assessment may capture only the initial wave of increase prior to the emergence of feedback loops, and hence underestimate population health benefit. Other interventions may have rapid impacts on outcomes which diminish, as an initially novel intervention washes out of its system over time. PokemonGo was discussed as a potentially game-changing physical activity intervention (LeBlanc et al., 2017), though engagement declined rapidly (Boulet, 2017). A short-term impact evaluation may have overestimated population health impacts. Changing one aspect of a complex social system may lead to actions to counter its effects by other groups with an interest in maintaining the status quo; immediate short-term effects of public health moves such as the forthcoming sugar tax in the UK for example might conceivably be nullified in the longer term via efforts of private sector producers to counter public health strategies.

Multiple follow-up measures may enable non-linearity of outcomes over time to be captured. However, except in cases where rich sources of routine data are available, resource requirements and research burden considerations may mean that only a small number of follow-ups is practical. This is particularly the case in RCTs, or studies with an unexposed control group. A systems lens however compels evaluators to justify decisions on when follow-ups should occur, grounded explicitly in a theorization of the, often non-linear, nature of outcomes over time. For psychological interventions, justifications for long-term follow-ups are sometimes framed on an assumption that short-term changes in behaviours will not be sustained (Simpson et al., 2015). However, because effects of more radical system changes may take time to build (Patton et al., 2006), evaluating outcomes only in the short-term risks the rejection of interventions which would have demonstrated effectiveness given more time. In evaluating the Learning Together school-based anti-bullying intervention, careful consideration was given to whether primary outcomes should be measured at two years, or whether changes to school environments would take longer than this to take effect; hence outcomes were measured at both two and three year follow-up (Bonell et al., 2014a).

Complex interventions in complex social systems pose almost infinite uncertainties (Moore et al., 2017), and there will always be much going on outside of the field of vision of an individual study. However, a focus on discrete impacts of system change does not necessarily betray a naïve view of how systems work, but may simply reflect a pragmatic focusing of research on core uncertainties. For example, introduction of smoke-free legislation gave rise to competing hypotheses regarding displacement of smoking into the home, or the de-normalization of smoking in front of children. The roots of these hypotheses are eminently compatible with a systems perspective, with their focus on how altering the dynamics of workplace settings may have knock on effects for how actors interact with other parts of the tobacco system. A systems lens was hence deployed strategically by an industry who used theorization of harmful unintended consequences as a means of instilling doubt regarding the merits of the ban (Chapman, 2007). The priority at this stage, rather than modelling the dynamics which may or may not give rise to these changes in fine detail, was to empirically test these concerns; a series of before and after studies was sufficient (Moore et al., 2012).

For many system changes, identifying appropriate outcomes may be challenging. Arguably, portrayals of RCTs as ‘gold standard’ and a tendency for funding panels to demand a single primary outcome has led to subversion of the theoretical origins of interventions to make them fit within this scientific paradigm (Sanson-Fisher et al., 2007). Settings approaches underpinning Health Promoting Schools (HPS) interventions for example emphasize the infeasibility of lots of single issue interventions in crowded school contexts – implementing one school-based intervention for smoking, one for physical activity, one for mental health and so on will never be sustainable – and hence highlight a need for a more holistic definition of health (Dooris, 2006). However, trials of HPS interventions remain focused on single issues, such as obesity or substance use (Langford et al., 2014, 2015), while more holistically defined settings based interventions have typically been evaluated within a more qualitative paradigm which frames outcomes as unknowable (Dooris, 2006). System changes which positively impact a range of outcomes, including for example cross-sectoral benefits on health and educational attainment, are likely to be more efficient and more sustainable in the longer term than those with a narrower focus. Hence, there is work to do in conceptualizing what effectiveness might look like in the context of interventions that focus on altering system characteristics which create the conditions to support a range of important outcomes for population health and beyond (Petticrew et al., 2017).

#### Evaluating process

One of the most fundamental changes within updated MRC guidance was recognition of the importance of process evaluation (Craig et al., 2008a). While the term ‘process’ is commonly seen as synonymous with qualitative methods, many early process evaluations involved purely quantitative implementation metrics (Oakley et al., 2006). These were largely used for the purpose of avoidance of type 3 error (Basch et al., 1985) through validating whether the intervention was delivered as intended, and hence, whether outcomes evaluation tested the a priori theory of change. Frameworks for process evaluation such as Steckler and Linnan’s (2002) focused firmly on efforts to quantify implementation, paying more limited attention to how the introduction of a new way of working served to disrupt system conditions. While necessary, quantitative assessments of implementation can only capture whether the anticipated changes took place. As described, responses of complex social systems to introduction of change are characterized by unpredictability (Chandler et al., 2016; Keshavarz et al., 2010; Rickles et al., 2007), which may give rise to a range of unintended emergent outcomes (Bonell et al., 2014b). Hence, limiting oneself to methods which capture only that which was anticipated in advance is problematic.

Newer guidance for process evaluation, though retaining a traditional definition of complex intervention focused primarily on their components, moved more explicitly toward a systems perspective. It emphasized the role of context in shaping the nature of any new intervention, the dynamic relationships between implementation, mechanisms and context, and the importance of understanding the temporally situated nature of process data in understanding the evolution of an intervention within its system (Moore et al., 2014, 2015a). It drew attention to the need to focus on concepts such as acceptability in dynamic terms, and to capture how system responses to interventions change over time as they generate positive and negative feedback loops. It moved toward a stronger emphasis on combining quantitative and qualitative research within process evaluation, with effective mixing of methods playing a vitally important role in capturing processes which were not anticipated, and modelling potential unintended consequences. When embracing a systems-based definition of fidelity focused on intervention function (Hawe et al., 2004), combining quantitative and qualitative methods is also vital in capturing how interventions are adapted across new contexts, and enabling judgements on the extent to which fidelity to function is maintained even where form varies (Van Urk, 2017).

As described, effects of any intervention are influenced strongly by the starting points of the system they attempt to disrupt. Hence, particularly for highly disruptive system change interventions like whole-school approaches, using evaluation to build and test theories about the functioning of systems and the processes of their disruption is vital (Bonell et al., 2018). Without this, the extent to which findings from evaluation will meaningfully inform practice in other spatial or temporal contexts, where educational, community and healthcare systems differ substantially, may always be limited. Of course, it is never possible to identify all potential system level mechanisms and moderators of the effects of an evaluation, and no evaluation would be powered to formally model all of these. However, combining quantitative causal modelling with qualitative process data can play a vital role in building and testing theories about the processes of disrupting the functioning of complex social systems to optimize their impacts on health.

As with earlier MRC guidance, the practice of researchers and funders in relation to process evaluation is likely to have begun to shift following the publication of new guidance; the role of process evaluation in understanding the processes associated with intervening within complex social systems may be increasingly realized in data emerging from recent evaluation. Further research including a number of the authors of the present article, and funded by MRC-NIHR, will also seek to provide guidance to researchers on how to adapt interventions which have shown effectiveness elsewhere for use in new social systems, and hence will aim to address some of the challenges in transferring evidence between systems identified here.

## Conclusions

Where viewing interventions as events within complex social systems, intervention development must begin with an understanding of the nature of the problem in the systems where intervention will take place. Broad-based evidence syntheses which focus on understanding the functioning of complex social systems, and drawing upon sources of interdisciplinary knowledge of the systems where change is proposed, is vital in developing interventions which work with the dynamics of the system to bring about positive change. Co-production with stakeholders at multiple positions within complex systems may be important in facilitating identification of system points which are modifiable, and have maximal leverage over system functioning. Intervention theories of change must consider not only what actions will be implemented, but what will be displaced, processes through which system changes will be achieved, and a range of scenarios about potential impacts on system functioning. Feasibility assessments, particularly of more ambitious system changes, then need to incorporate dynamic and explicitly temporal dimensions to understand how interventions have the potential to become integrated into their systems over time. A whole systems approach to evaluation may never be achievable; introduction of changes to complex social systems will always give rise to more uncertainties than a single evaluation can satisfactorily capture. However, the added value of a system lens for evaluation perhaps lies in ensuring that evaluation focuses on the most important areas of uncertainty to move intervention science forward, and in justifying decisions such as the length of time at which assessment of impact can be expected to be meaningful. Key areas for further methodological debate and development in exploring the potentials of a systems perspective include: i) examples of the use of systems science methods in the development of interventions in complex social systems, ii) greater consideration of how to operationalize ‘feasibility’ of proposed changes to complex social systems, and develop criteria for decision making regarding progression to full evaluation in the context of ambitious system changes, and iii) methods for making judgements of the effectiveness of whole-system changes which are not easily evaluable via a focus on discrete health outcomes, and better use of process evaluation data to build theory on the processes of changing system functioning in order to inform judgements on the transferability of evidence between systems.

## Notes

1.
https://www.nihr.ac.uk/funding-and-support/documents/funding-for-research-studies/research-programmes/PGfAR/CCF-PGfAR-Feasibility-and-Pilot-studies.pdf
